# High-throughput sequencing analysis revealed the regulation patterns of small RNAs on the development of *A. comosus* var. *bracteatus* leaves

**DOI:** 10.1038/s41598-018-20261-z

**Published:** 2018-01-31

**Authors:** Ying-Yuan Xiong, Jun Ma, Ye-Hua He, Zhen Lin, Xia Li, San-Miao Yu, Rui-Xue Li, Fu-Xing Jiang, Xi Li, Zhuo Huang, Ling-Xia Sun

**Affiliations:** 1 College of Landscape Architecture of Sichuan Agricultural University, Chengdu, Sichuan 611100 China; 2 Horticultural Biotechnology College of South China Agricultural University, Guangzhou, Guangdong 510642 China

## Abstract

Studies of the molecular mechanisms involved in the formation of the albino leaf cells are important for understanding the development of chimera leaves in *Ananas comosus* var. *bracteatus*. In this study, we identified a total of 163 novel miRNAs involved in the development of complete white (CWh) and complete green (CGr) leaves using high-throughput sequencing method. The potential miRNA target genes were predicted and annotated using the NR, Swiss-Prot, GO, COG, KEGG, KOG and Pfam databases. The main biological processes regulated by miRNAs were revealed. The miRNAs which potentially play important roles in the development of the leaves and the albino of the CWh leaf cells were selected and their expression patterns were analyzed. The expression levels of nine miRNAs and their potential target genes were studied using qRT-PCR. These results will help to elucidate the functional and regulatory roles of miRNAs in the formation of the albino cells and the development of the leaves of *A. comosus* var. *bracteatus*. These data may also be helpful as a resource for studies of small RNA in other leaf color chimeric plant species.

## Introduction

*Ananas comosus* var. *bracteatus* (red pineapple) is a commercially cultivated monocot plant originating from South America. It belongs to the *Bromeliaceae* family. While other plants in this family are valued for their fruit, the high quality silk fiber of the stem and leaves^1,2^, and a large number of secondary metabolites^3–8^, the red pineapple is cultivated commercially as an ornamental plant because of its colorful chimera leaves. The chimera leaves consist of normal green cells and albino white cells, but the chimeric character of the leaves in cultivated plants is particularly unstable. The leaf color of the regenerated plants varies significantly during tissue culture, in which only about 1% of the regenerated plants have the same chimeric character as the mother plant. Most of the regenerated plants lose their economic value due to the lack of colorful chimera leaves. In order to understand the molecular mechanism of the formation of the albino cells and the development of the leaves, we undertook transcriptome sequencing of the CGr cells and the CWh cells. The results of transcriptome sequence revealed the significant differences in transcription levels between the CGr and CWh plants, especially in those aspects that control photosynthesis, porphyrin and chlorophyll metabolism, and carotenoid biosynthesis^9^. These studies provided a frame of transcription variations between CGr and CWh plants that most likely result in the formation of the albino leaf cells. Based on these results, we could further study the regulatory roles of small RNA presumably involved in the development of albino leaves.

MicroRNAs are the short non-coding RNAs (20–24 nucleotides) that silence the expression of target genes through complementary sequence matching^10^. In plants, miRNAs control the expression of various genes involved in the processes of development, growth, and physiology. In particular, these include genes that encode transcription factors, stress response proteins, and other proteins^11^. Although the regulation of genes by miRNA is indirect, it is believed that miRNAs play important roles in plant growth and development^12^.

In this study, small RNA libraries were constructed at the different developmental stages of CWh and CGr leaves. 163 novel miRNAs and their potential target genes were identified by Illumina sequencing. To our knowledge, this is the first comparative miRNA high-throughput sequencing of *A. comosus* var. *bracteatus*. This study was mainly focused on revealing the expression profiles and regulation patterns of miRNAs involved in the CGr and CWh leaf development. The miRNAs which play potentially important roles in the development of the leaves and the albino of the CWh leaf cells were selected and analyzed. The expression levels of nine miRNAs and their potential target genes were detected by real-time qPCR. Our results provide a valuable resource for studying microRNA regulation involved in the leaf color formation and leaf development of *A. comosus* var. *bracteatus*.

## Materials and Methods

### Plant material

**Figure 1:**
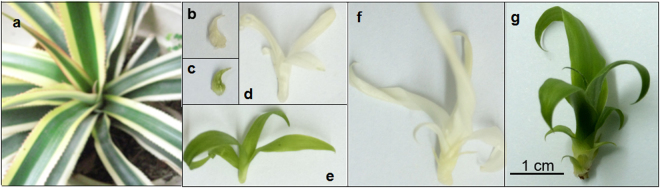
Materials for miRNA sequence. (**a**) Wild chimeric plant of *A. comosus* var. *Bracteatus*. (**b**) Complete white shoots at developmental stage 1 (with unexpanded leaves, WS1). (**c**) Complete green shoots at developmental stage 1 (GS1). (**d**) Complete white shoots at developmental stage 2 (with four to five expanded leaves, WS2). (**e**) Complete green shoots at developmental stage 2 (GS2). (**f**) Complete white shoots at developmental stage 3 (with ten to twelve expanded leaves, WS3). (**g**) Complete green shoots at developmental stage 3 (GS3).

Complete green (GS1, GS2, GS3) and white (WS1, WS2, WS3) shoots were derived from the stem explants of chimera plants of *A. comosus* var. *bracteatus* (Fig. 1a) by tissue culture, in accordance with our previous protocol. Three developmental stages were evaluated based on the number of expanded leaves on shoots. WS1 (Fig. 1b) and GS1 (Fig. 1c) are complete white and green shoots respectively with only unexpanded leaves; WS2 (Fig. 1d) and GS2 (Fig. 1e) are complete white and green shoots respectively with four to five expanded leaves; WS3 (Fig. 1f) and GS3 (Fig. 1g) are complete white and green shoots respectively with ten to twelve expanded leaves. The leaves were collected at the above three stages, immediately frozen in liquid nitrogen, and then stored at −80 °C until analysis.

### Measurement of chlorophyll and carotenoid contents

Complete green (GS1, GS2, GS3) and complete white (WS1, WS2, WS3) leaves were selected for chlorophyll and carotenoid measurements. The contents of chlorophyll and carotenoid concentrations were measured using the Holm equation and the method as previously described^13^.

### RNA extraction, small RNA library construction and sequencing

Total RNA was isolated from six samples using Trizol (Invitrogen, USA) according to the manufacturer’s instructions. The purity, concentration and integrity of RNA samples was analyzed using Nanodrop, Qubit 2.0, the Agilent 2100 bioanalyzer respectively in order to ensure the high quality of sample for sequencing. A total amount of 1.5 μg RNA per sample was used as input material for the RNA sample preparations. Sequencing libraries were generated using NEB Next Ultra small RNA Sample Library Prep Kit for Illumina (NEB, USA) following manufacturer’s recommendations and index codes were added to attribute sequences to each sample. The clustering of the index-coded samples was performed on a cBot Cluster Generation System using TruSeq PE Cluster Kit v4-cBot-HS (Illumia) according to the manufacturer’s instructions. After the cluster generation, the library preparations were sequenced on an Illumina Hiseq 2500 platform and single-end 50 nt reads were generated (Beijing Biomarker Technologies Co., Ltd, Beijing, China).

### Identification and bioinformatics analysis of miRNAs

Raw data (raw reads) of fastq format were firstly processed through in-house perl scripts. In this step, clean data (clean reads) were obtained by removing reads containing adapter, reads containing ploy-N and low quality reads from raw data. And reads were trimmed and cleaned by removing the sequences smaller than 18 nt or longer than 30 nt. Using Bowtie tools soft, the Clean Reads were aligned respectively with Silva database, GtRNAdb database, Rfam database and Repbase database to filter ribosomal RNA (rRNA), transfer RNA (tRNA), small nuclear RNA (snRNA), small nucleolar RNA (snoRNA) and other ncRNA and repeats. The clean reads were mapped to genomic sequences (Acomosus_321_v3, https://phytozome.jgi.doe.gov/pz/portal.html#!info?alias=Org_Acomosus_er). The miRDeep2^14^ was used to detect the known miRNA and new miRNA by comparing with known miRNAs from miRBase.

### Quantification of miRNA expression levels and identification of differentially expressed miRNA

miRNA expression levels were estimated for each sample: sRNA were mapped back onto the precursor sequence. Read count for each miRNA was obtained from the mapping results. The differentially expressed miRNA was identified by IDEG6^15^. The rules used for identification of differentially expressed miRNA were |log2(FC)| > = 1 and FDR < = 0.01.

### Prediction of miRNA target genes and function annotation

The potential target genes of sequenced miRNAs were predicted by Target Finder^16^. Gene function was annotated based on the following databases: Nr (NCBI non-redundant protein sequences)^17^; Nt (NCBI non-redundant nucleotide sequences); Pfam (Protein family)^18^; KOG/COG (Clusters of Orthologous Groups of proteins)^19,20^; Swiss-Prot (A manually annotated and reviewed protein sequence database)^21^; KO (KEGG Ortholog database); GO (Gene Ontology)^22^ and KEGG (http://www.genome.jp/kegg/kaas/) database.

### qRT-PCR identification of the expression levels of miRNAs and potential target genes

Total RNAs of six samples as used in transcriptome sequence were reverse transcribed into cDNA using the TURE script 1st Stand cDNA SYNTHESIS Kit (Aidlab) with miRNA specific primer (Table S1). qRT-PCR was performed using the analytikjena-qTOWER2.2 Real-Time PCR System (Germany) with SYBR® Green (DBI). PCR reactions of miRNAs were performed as follow: 95 °C for 3 min, followed by 40 cycles of 95 °C for 10 s, and 58 °C for 30 s. *U6* gene was used as internal control. PCR reactions of potential target genes were performed at 95 °C for 3 min, followed by 40 cycles of 95 °C for 10 s and 58 °C for 30 s. A melting curve (at 60–95 °C) was generated to check amplification specificity. Each reaction presented three biological repeats. The primers used were listed in Table S2.

## Results

### Chlorophyll and carotenoid content analysis of the CWh and CGr leaves among three developmental stages

**Figure 2:**
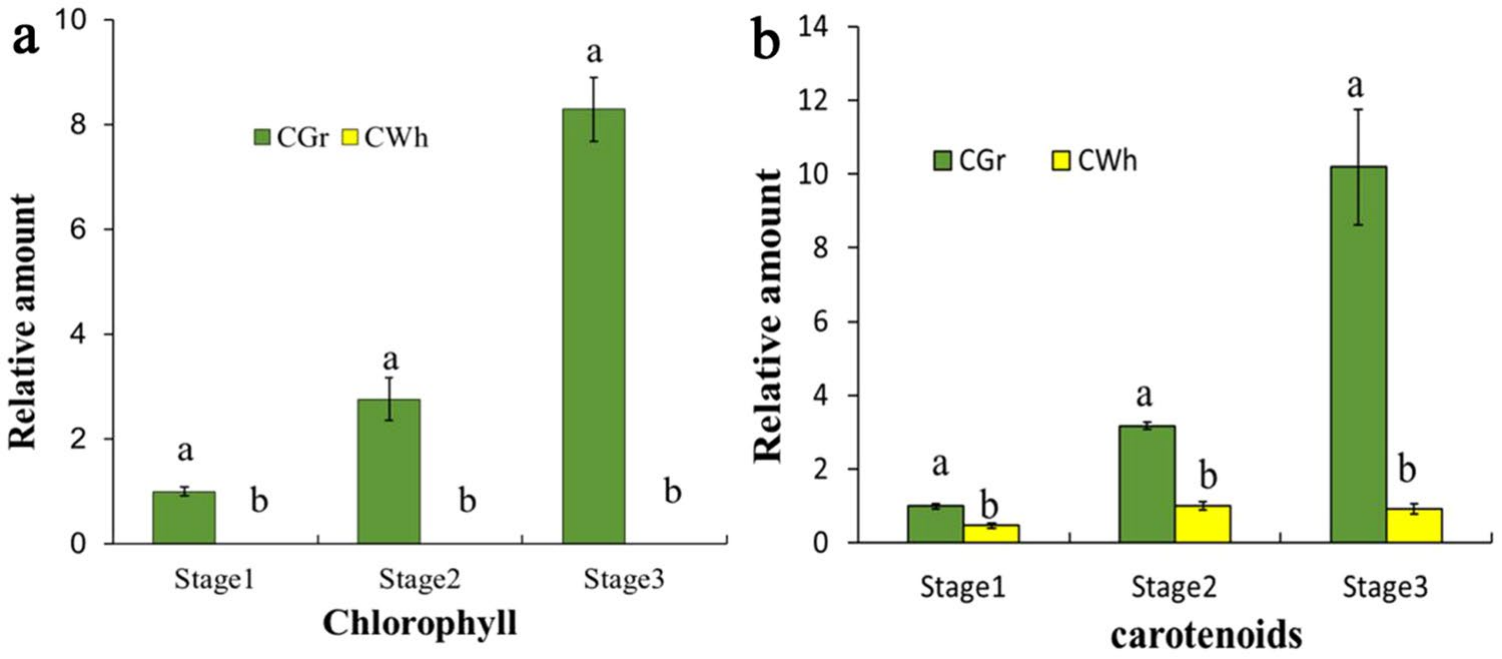
Pigment concentrations of the CGr and CWh leaves. (**a**) The concentration of chlorophyll in the CGr and CWh leaves at three developmental stages. (**b**) The concentration of carotenoids in the CGr and CWh leaves at three developmental stages. The relative values of pigments concentrations were calculated using the value of CGr leaves at developmental stage 1 as 1. Different letters in columns indicate statistically significant differences (P < 0.01) according to a T-test.

The content of chlorophyll in CWh leaves is not detectable among all three developmental stages, while that in CWh leaves is significantly high, and increases gradually with the development of plants. (Fig. 2). These results confirmed that the CWh and CGr leaves were the typical presentations of the albino white cells and the normal green cells, respectively. The content of carotenoid of the CGr leaves is greatly higher than that in the CWh leaves among all three developmental stages, and it is gained gradually in both CGr and CWh leaves along the development of the plants (Fig. 2).

### High-throughput small RNA sequencing of *Ananas comosus* var. *bracteatus* leaves

Our former studies had confirmed that chlorophyll in CWh leaves is significantly suppressed, and that the transcriptional pattern of CWh leaves is different from that of CGr leaves^9^. In order to identify miRNAs and to reveal the regulation mechanisms of gene expression in the *A. comosus* var. *bracteatus* leaves, the high-throughput sequencing of six miRNA libraries constructed from the CGr and CWh leaves at three developmental stages were performed. After filtering the low-quality reads, we obtained 16–18 million clean reads from each sample (Table S3). Among them, 5.8–7.4 million (about 35–45%) reads were perfectly mapped to the *A. comosus*_321_v3 genome. All of the clean reads mapped to the *A. comosus*_321_v3 genome were used for miRNA identification by miRDeep2. To facilitate the access and use of the sequence data, the data have been submitted to NCBI with BioProject number: PRJNA389361.

The different types of total small RNA sequences were further classified after using the Bowtie software to blast the clean reads against the Silva, GtRNAdb, Rfam and Repbase databases^23^. The clean reads were annotated as tRNAs, rRNA, snRNA, scRNA, snoRNAs, and repeat-associated small RNAs based on the Rfam database (Table S4).

### Identification of miRNAs in *A. comosus* var. *Bracteatus*

All small RNA sequences were aligned to the precursor/mature miRNAs in the miRbase database using miRDeep2 software^24^. No known-miRNA was predicted. A total of 162, 163, 162, 162, 163, 161 novel miRNAs were identified in the six samples, respectively. The sequences of the 163 miRNAs are listed in Table S5. Among the 163 miRNAs, 104 miRNAs are conservative and 59 miRNAs are un-conservative. The length distribution of the identified miRNAs was shown in Fig. S1a. The most abundant sequence size is 21 nt-long, followed by 24 nt. The base bias on the first site of miRNAs with specific lengths and a specific site of miRNAs were shown in Fig. S1b,c. The majority of the 163 miRNAs started with a 5′-U, except for the 24nt-long miRNAs, which mostly started with A (59.26%). All the 19 nt-long miRNAs started with a 5′-U, followed by 21 nt-long miRNA (70.31%). The (A + U) percentage of the first nucleotide bias of 21 nt-long and 24-long miRNAs is 85.94% and 75.93% respectively. Nucleotide bias analysis at each position indicated that (A + U) occupied a very high percentage (77.16%) at the start of reads, while (G + C) occupied high frequency (61.02% at 24 nt and 80% at 25 nt) at the ends of reads. The distribution of the 163 identified miRNAs in each miRNA family was shown in Fig. S2. The 163 miRNAs were predicted to belong to 46 miRNA families. The most enriched family is miR399 (10), followed by miR159 (6), miR160 (6), miR164 (6) and miR166 (5).

### Prediction and annotation of miRNA putative target genes

The putative target genes of miRNA were predicted by Target Finder software. Among the 163 miRNAs, 123 miRNAs were predicted with 488 potential target genes. The potential target genes were blasted to the Nr, Nt, Swiss-Prot, GO, COG, KEGG, KOG and Pfam databases. The statistic of the functional annotation of the target genes were listed in Table S6.

The Nr homologous species distribution of the 414 candidate target genes were shown in Fig. S3a. The blasted species are all monocots. The first three enriched species are *Elaeis guineensis* (166), *Phoenix dactylifera* (123) and *Musa acuminate* (42), which are all perennial tropical monocots. The blast results of the potential target genes found in COG database were shown in Fig. S3b. A total of 155 target genes were assigned to COG classification. The target genes were classified into 25 categories. The largest group is ‘General function prediction only’, followed by ‘Transcription’ and ‘Replication, recombination and repair’, and then ‘Signal transduction mechanisms’. GO analysis was conducted to describe the functional classification of target genes in terms of their associated biological processes, cellular components, and molecular functions. A total of 243 target genes were annotated by GO database (Fig. S3c). ‘Cell part’, ‘Cell’ and ‘Organelle’ are the most enriched groups under the cellular component category. The most highly represented groups of molecular function category are ‘Binding’ and ‘Catalytic activity’. For the biological process category, the ‘Metabolic process’, ‘Cellular process’ and ‘Single-organism process’ are the most enriched groups. The potential target genes were also blasted to the KEGG database using BLASTx. Forty-two KEGG pathways were annotated by the target genes (Fig. S3d). The most frequently represented pathways are ‘Biosynthesis of amino acids’ (ko01230, 5), ‘Oxidative phosphorylation’ (ko00190, 5), followed by ‘Tropane, piperidine and pyridine alkaloid biosynthesis’ (ko00960, 4) and ‘mRNA surveillance pathway’ (ko03015, 4).

### Expression profiles of the miRNAs in different samples

**Figure 3:**
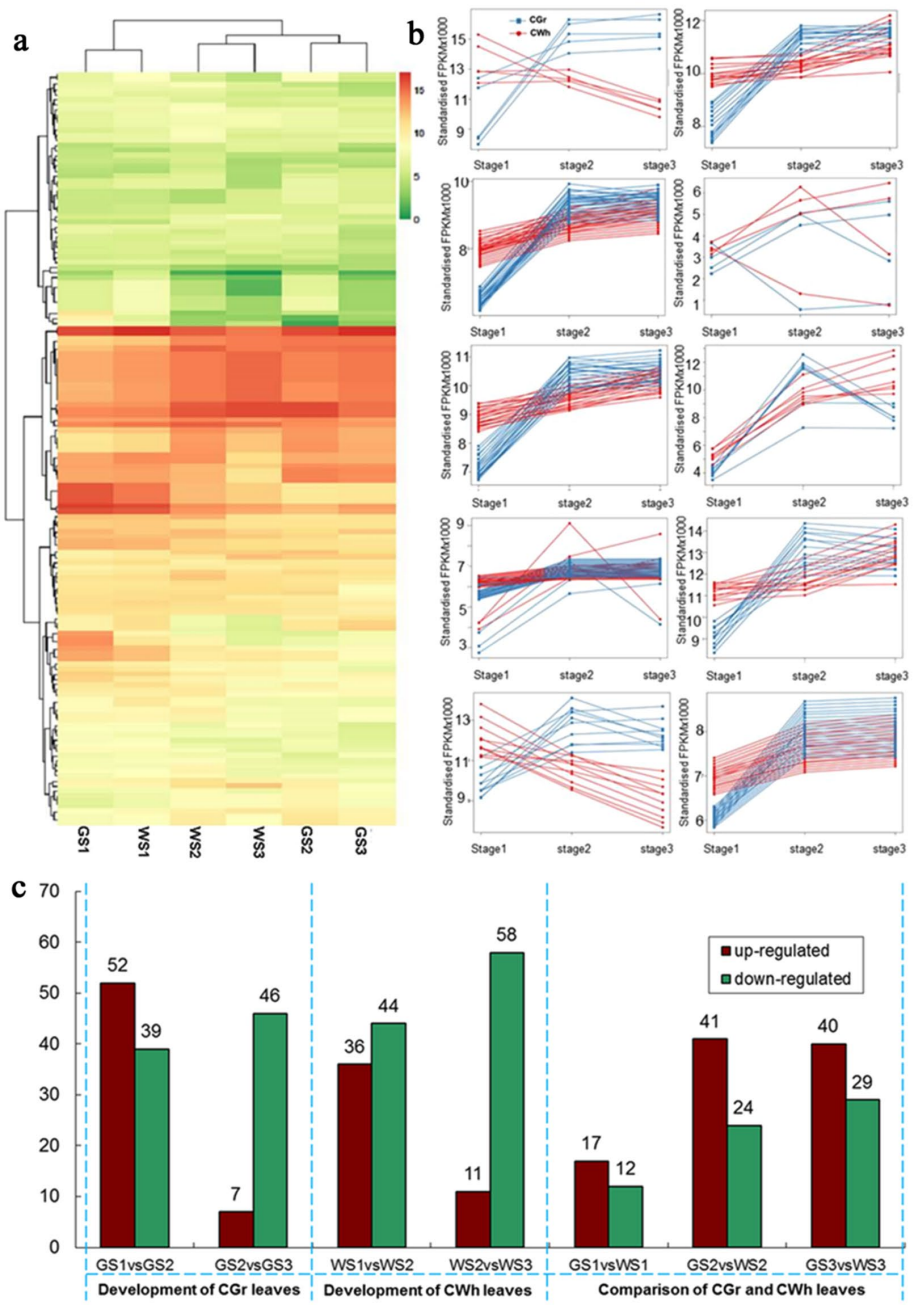
Expression and clustering analysis of the miRNAs. (**a**) Heat map of differentially expressed miRNAs detected in this sequence based on their expression in 6 samples. (**b**) Cluster analysis of miRNAs with the K-means method. (**c**) Comparison of the number of differentially expressed miRNAs in the six samples. *Development of CGr leaves*: number of differentially expressed miRNAs in CGr leaves at three developmental stages. *Development of CWh leaves*: number of differentially expressed miRNAs in CWh leaves at three developmental stages. *Comparision of CGr and CWh leaves*: number of differentially expressed miRNAs between CGr and CWh leaves at three developmental stages. *Up-regulated*: the expression level of the miRNA is up-regulated in the latter sample compare to the former sample in two compared samples. *Down-regulated*: the expression level of the miRNA is down-regulated in the latter sample compare to the former sample between two compared samples.

The expression levels of the 163 miRNAs in the six samples were digitally measured and their expression relationships were characterized. The expression levels of the miRNAs were analyzed through the heat map method (Fig. 3a). The six samples were clustered to three groups, GS1 and WS1, WS2 and WS3, GS2 and GS3. Ten clusters were obtained from the expression data of the six samples using K-means method (Fig. 3b). These miRNAs target on genes functioned in transcription and translation, pigment biosynthesis and chloroplast development, sulfate assimilation, starch synthase, disease resistance ect. These miRNAs may presumably play very important roles in the growth and development of the complete white leaves.

The statistics of the number of differentially expressed miRNAs was shown in Fig. 3c. There are total 91 and 80 differentially expressed miRNAs between stage 1 and stage 2, in the CGr and CWh leaves respectively and about half of the differentially expressed miRNAs is down-regulated. Moreover, there are total 53 and 69 differentially expressed miRNAs between stage 2 and 3 in the CGr and CWh leaves respectively, and 87% and 84% of the differentially expressed miRNAs are down-regulated between stage 2 and 3 in CGr and CWh leaves respectively. This indicated that the number of differentially expressed miRNAs is decreased along the development of the plants in both CGr and CWh leaves. Compared with CGr and CWh leaves, there are 29, 65, 69 differentially expressed miRNAs at stage 1, 2, 3 respectively. At stage 2 and stage 3, the numbers of up-regulated (41, 40) and down-regulated miRNAs (24, 29) among CGr and CWh leaves are similar.

In order to identify potentially important miRNAs which functioned in the albino and development of the CWh leaves, the differentially expressed miRNAs (|log2 (FC)| ≥ 3 and FDR ≤ 0.01) between CGr and CWh leaves are selected and analyzed (Table 1). The expression of all the miRNAs except *Ab*-miR131, *Ab*-miR132, *Ab*-miR139 and *Ab*-miR152 are down regulated in CWh leaves. It is worth mentioning that the expression values of the differentially expressed miRNAs between CGr and CWh at three developmental stages are different, except *Ab*-miR7. The log2FC (WS/GS) valued of *Ab*-miR7 is −3.84 and −5.83 at developmental stages 2 and 3, respectively. *Ab*-miR7 is predicted to target on genes play roles in photosynthesis, sulfur metabolism, non-homologous end-joining and translation initiation.

**Table 1:** Differentially expressed miRNAs (| log2 (FC) | ≥ 3 and FDR ≤ 0.01) between CGr and CWh leaves.

Stage	MiRNA	Family	Target gene functions	log2FC(WS/GS)
GS1vsWS1	*Ab*-miR45	MIR1319	SNARE interactions in vesicular transport Oxidative phosphorylation	−3.23
	*Ab*-miR84		Endocytosis	−4.08
	*Ab*-miR106	MIR482	Transcription Protein phosphorylation Development and cell death	−3.33
	*Ab*-miR107	MIR482	Same as *Ab*-miR106	−3.33
	*Ab*-miR124		Biosynthesis of amino acids Photosynthesis Response to stress	−3.44
GS2vsWS2	*Ab*-miR6	MIR395	Photosynthesis Sulfur metabolism Non-homologous end-joining Translational initiation	−3.03
	*Ab*-miR7	MIR395	Same as *Ab*-miR6	−3.48
	*Ab*-miR46	MIR399	Plant hormone signal transduction	−3.72
	*Ab*-miR152	MIR1120	Transcription factor bHLH	3.19
	*Ab*-miR144	MIR399	rRNA methylation	−3.45
	*Ab*-miR139	MIR1516	No target gene predicted	3.31
GS3vsWS3	*Ab*-miR7	MIR395	Same as *Ab*-miR6	−5.83
	*Ab*-miR8	MIR395	Sulfur metabolism Signal transduction Leaf development Translational initiation Non-homologous end-joining	−4.66
	*Ab*-miR11	MIR528	Oxidation-reduction process Starch synthase Phosphorylation Endocytosis	−3.19
	*Ab*-miR27	MIR528	Same as *Ab*-miR11	−3.25
	*Ab*-miR101	MIR528	Same as *Ab*-miR11	−3.25
	*Ab*-miR131	MIR171_2	No target gene predicted	3.24
	*Ab*-miR132		No target gene predicted	3.62

### Expression patterns of miRNAs targeted genes related to transcription factors

The expression patterns of the miRNAs target on transcription factors were shown in Table 2. There are fifteen miRNAs target on twelve transcription factors. Among them, *Ab*-miR152 and *Ab*-miR39 are differentially expressed between CGr and CWh leaves at all three developmental stages. *Ab*-miR87 and *Ab*-miR124 are differentially expressed between CGr and CWh leaves at only developmental stage 1 and 2.

**Table 2:** The expression patterns of the miRNAs targeted genes related to transcription factors.

DEG ID	Target gene annotation	GS1vsWS1	GS2vsWS2	GS3vsWS3	GS1vs GS2	GS2vs GS3	WS1vsWS2	WS2vsWS3
*Ab*-miR73 *Ab*-miR90	AP2-like ethylene-responsive transcription factor	— —	−*2.95* —	— —	*1.84* —	— —	— —	*1.49* —
Ab-miR37, Ab-miR152, Ab-miR52	bHLH49 isoform X1 bHLH18-like bHLH140-like	— *3.19* —	— *2.26* —	*1.06* *−2.22* —	— — —	— — *−1.34*	— — *1.09*	— *−1.86* *−1.48*
*Ab*-miR37	GAMYB-like isoform X2	—	—	*1.06*	—	—	—	—
*Ab*-miR68	homeobox-leucine zipper protein	—	—	*1.20*	*2.46*	−1.23	1.68	—
*Ab*-miR52, *Ab*-miR87	WRKY48 WRKY9	— 1.82	— −2.32	— —	— 1.29	−1.34 −4.10	1.09 *−2.84*	−1.48 −1.74
*Ab*-miR9	CCAAT-binding transcription factor	—	—	−1.62	*−1.92*	—	*−1.62*	—
*Ab*-miR18	TCP family transcription factor	—	*1.46*	—	*−3.83*	—	*−2.04*	—
*Ab*-miR94	LIM domain-containing protein WLIM1	—	—	*2.32*	1.89	−2.17	*_*	1.04
*Ab*-miR49	Transcription factor BRX N-terminal domain	*_*	1.16	—	—	—	—	−1.60
*Ab*-miR39	SRF-type transcription factor	*1.60*	−2.25	−1.09	1.49	−2.75	−2.36	−1.59
*Ab*-miR124	Trihelix transcription factor GT-3b	*−3.44*	−2.37	—	−6.09	—	−5.02	—
*Ab*-miR156	MADS-box transcription factor 50	—	2.13	—	−1.65	—	—	−1.91

Note: the values in each column (AvsB) was the value of [log2FC (B/A)].

— means no significant differences were detected between the two samples.

### Identification of miRNAs target on genes related to chlorophyll biosynthesis and photosynthesis

The differentially expressed miRNAs with target genes related to chlorophyll biosynthesis and photosynthesis are listed in Table 3. There are seven miRNAs predicted to target on chlorophyll biosynthesis and nine miRNAs targeted on photosynthesis. *Ab*-miR73, *Ab*-miR90 and *Ab*-miR98 are predicted to target on Ferrochelatase (HemH, EC: 4.99.1.1), and are down regulated in CGr leaves at developmental stage 2. *Ab*-miR127, *Ab*-miR160, *Ab*-miR161 and *Ab*-miR163 are targeted on divinyl chlorophyllide a 8-vinyl-reductase (DVR, EC: 1.3.1.75), which are down regulated at stage 1 and up regulated at stages 2 and 3 in CWh leaves. And these four miRNAs are down regulated at developmental stage 2 compare to developmental stage 1 in both CGr and CWh leaves. *Ab*-miR6, *Ab*-miR7 and *Ab*-miR8 targeted on PGR-5 like protein (Ferredoxin-plastoquinone reductase 1), which is involved in the cyclic electron flow (CEF) around photosystem I^25^. *Ab*-miR6, *Ab*-miR7 and *Ab*-miR8 are up regulated at stage 1 and down regulated at stage 2 and stage 3 in CWh leaves. *Ab*-miR124 is predicted to target on trihelix transcription factor, which may act as a molecular switch in response to light signals^26^, and which is significantly down regulated in CWh leaves at developmental stage 1 and 2. *Ab*-miR124 is significantly down regulated along the development of both CGr and CWh leaves. The other miRNAs listed are targeted on genes related to the reaction center of the photosynthesis system^27–29^.

**Table 3:** The expression levels of differentially expressed miRNAs with target genes related to chlorophyll biosynthesis and photosynthesis.

DEG ID	Target gene annotation	GS1vsWS1	GS2vsWS2	GS3vsWS3	GS1vsGS2	GS2vsGS3	WS1vsWS2	WS2vsWS3
*Ab*-miR127 *Ab*-miR160 *Ab*-miR161 *Ab*-miR163	divinyl chlorophyllide a 8-vinyl-reductase	*−1.10* *−1.10* *−1.10* *−1.10*	*2.06* *2.06* *2.06* *2.06*	*1.25* *1.25* *1.25* *1.25*	*−5.32* *−5.32* *−5.32* *−5.32*	— — — —	*−2.16* *−2.16* *−2.16* *−2.16*	— — — —
*Ab-*miR73 *Ab-*miR90 *Ab-*miR98	Ferrochelatase	—	*−2.95* *−2.95* *−2.95*	—	*1.84* *1.84* *1.84*	— — —	— — —	*1.49* *1.49* *1.49*
*Ab*-miR6 *Ab*-miR7 *Ab*-miR8	PGR5-like protein	*1.35* *1.32* 1.39	*−3.03* *−3.48* −2.17	−5.50 −5.83 −4.66	— *1.03* —	*1.04* *1.15* *1.02*	*−3.58* *−3.76* *−3.64*	*−1.42* *−1.20* *−1.47*
*Ab*-miR124	Trihelix transcription factor	*−3.44*	*−2.37*	—	*−6.09*	—	−5.02	—
*Ab*-miR52 *Ab*-miR83	*PsbB (CP47)* tetrapyrrole-binding protein	— —	— —	— *1.60*	— —	−1.34 −1.36	1.10 —	−1.48 —
*Ab*-miR108	Ferredoxin-thioredoxin reductase	—	1.16	—	—	—	—	*−1.17*
*Ab*-miR82 *Ab*-miR104	*PsbP* sedoheptulose-1,7-bisphosphatase	— —	— —	1.65 1.65	*−3.19* *−3.19*	*−1.29* *−1.29*	*−2.08* *−2.08*	— —
*Ab*-miR3	Chlorophyll a-b binding protein	—	—	1.51	−1.55	−1.19	—	—
*Ab*-miR101	polyphenol oxidase	—	−1.66	−3.19	1.11	—	—	−1.77
*Ab*-miR11	polyphenol oxidase granule-bound starch synthase 1	—	−1.66 −1.67	−3.19 −3.25	1.11 1.10	— —	— —	−1.77 −1.81

Note: the values in each column (AvsB) was the value of [log2FC (B/A)].

— means no significant differences were detected between the two samples.

### Expression profiles of nine miRNAs and their potential target genes

**Figure 4:**
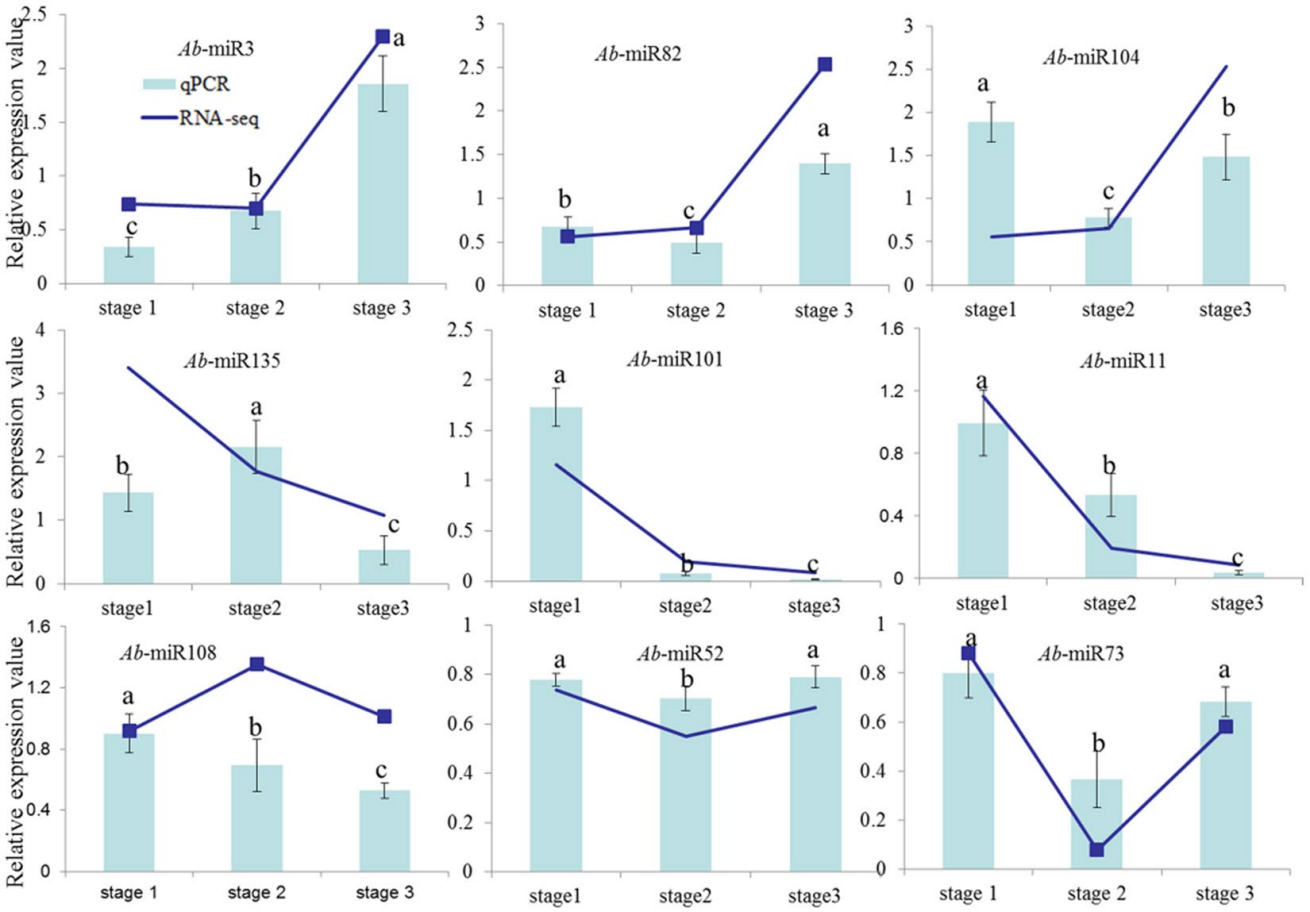
Expression profiles of miRNAs in the CGr and CWh leaves at three developmental stages. The relative expression values were the value of CWh/CGr. Each bar represents the mean value from triplicate experiments ±SD. The letters marked on the columns indicate statistically significant differences (*P* < 0.05) according to a T-test.

**Figure 5:**
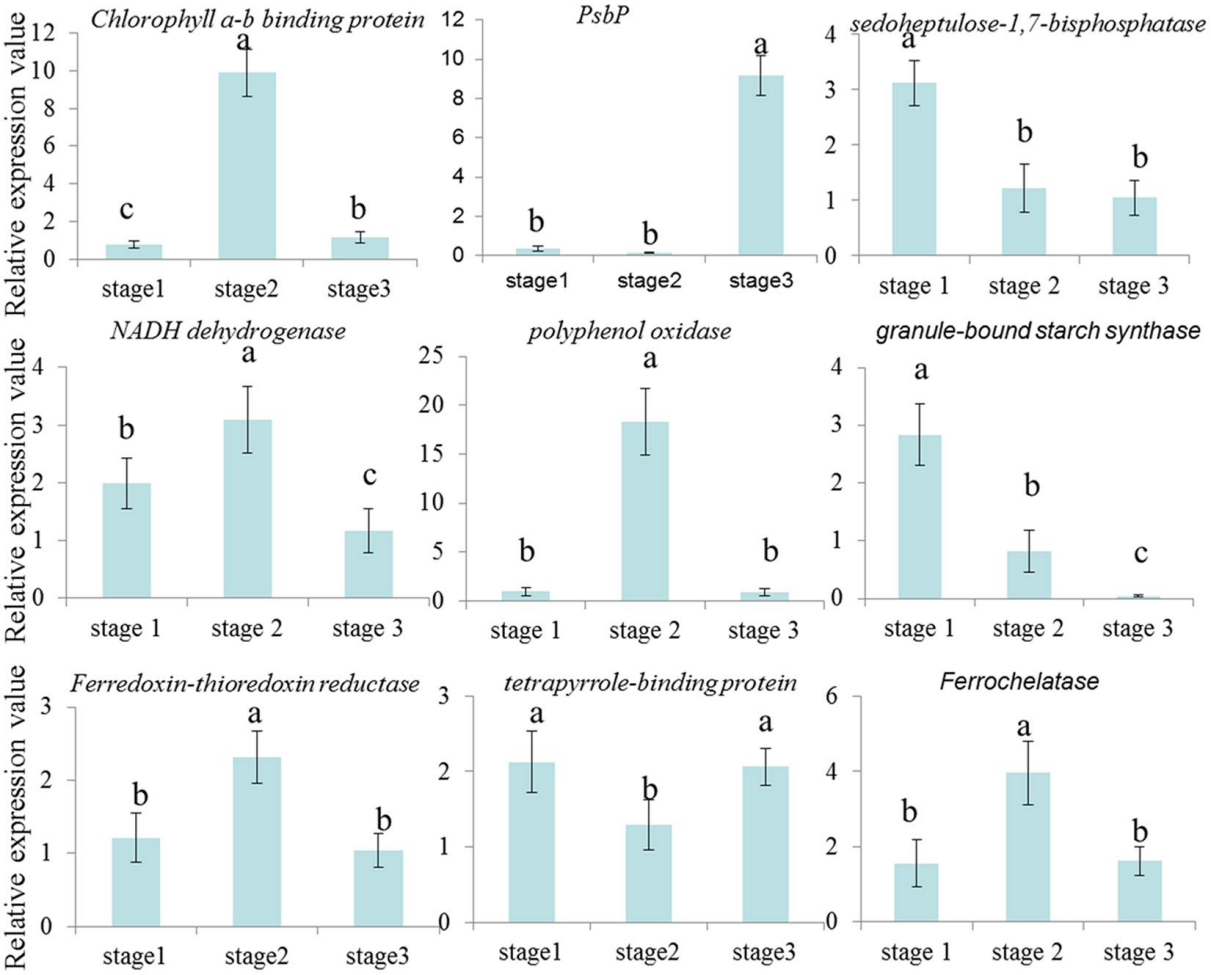
The expression patterns of the potential target genes at three developmental stages. The relative expression values were the value of CWh/CGr. Each bar represents the mean value from triplicate experiments ±SD. The letters marked on the columns indicate statistically significant differences (*P* < 0.05) according to a T-test.

In order to reveal the potential regulatory mechanism of the miRNAs action on the target genes, the expression profiles of nine miRNAs presumably relating to photosynthesis and chlorophyll biosynthesis, and their potential target genes were detected using qRT-PCR. The expression patterns of these miRNAs detected by RNA-seq are well corroborated by qRT-PCR (Fig. 4). This confirmed that the sequence results are reliable. The expression levels of the nine potential target genes of the nine miRNAs analyzed by qRT-PCR are shown in Fig. 5. Our results showed that the expression levels of these target genes are not all dependent on their predicted miRNAs based on the analysis of their expression levels. Many factors may affect the accuracy of target gene prediction and the regulatory relationships between miRNA and their target genes. For instance, the lacking of relevant research and genomic information may affect the accuracy of target gene prediction^30^. It is the first report of miRNA sequencing in *A. comosus* var. *bracteatus*, and the identification of miRNA is based on the genomic information of *A. comosus* var. *comosus*. Furthermore, some of these potential target genes are predicted to be regulated by more than one miRNAs at the translational level^30^. It is reported that isomeric sequences shared the authentic miRNA target genes with canonical miRNA^31^.

## Discussion

The unstable chimeric character of the leaves in *A. comosus* var. *bracteatus* during tissue culture inhibits the commercial production of clones through tissue culture. Therefore, elucidating the albino mechanism of leaf cells is significant on the regulation of the stability of the chimera character in this plant. It is well known that miRNAs play important regulatory roles in gene expression at the post-transcriptional level by repressing the gene translation^32^. Previous studies have confirmed that miRNAs play a role in leaf development in higher plants^32–34^. It is reported that miRNAs are involved in regulation of many developmental processes, such as meristem identity, cell division and organ separation^35–37^.

In this study, we identified miRNAs from CGr and CWh leaves at three development stages by high-throughput sequencing. A total of 163 miRNAs were identified. These miRNAs belong to 46 miRNA families. The top five enriched miRNA families are miR399, miR159, miR160, miR164 and miR166. miR399 is involved in functions which respond to nutrient deficiency^38,39^ and are also an important regulator of Pi starvation-induced root-associated acid phosphatase activity^40^. miR159 negatively controls the expression of MYB33 and MYB65^41,42^, and the expression of miR159 is suppressed by DELLAs^43^. The miR159 works on leaf development and cell division by targeting TCP transcription factor genes^44,45^. The miR160 targets the auxin response factors (ARFs) to negatively regulate the auxin signaling^46–48^. The miR164 targets the NAC transcription factor ORE1^49^ and CUC^50^, both involved in functions related to leaf development. The miR 164 also functions in response to hormone signals by targeting the NAC genes^50,51^. The miR166 regulates shoot apical meristem splitting by targeting the class III homeodomain leucine zipper transcription factors^52^. These results indicated that these growth and development aspects of *A. comosus* var. *bracteatus* may be regulated by miRNAs.

Along the development of both the CGr and CWh leaves, the number of differentially expressed miRNAs decreased. And the expression levels of about 85% of the differentially expressed miRNAs are decreased at development stage 3 compared to development stage 2. This data suggested that the regulation function of miRNA is more important at the early developmental stages of the leaves.

Since the expression levels of the seven miRNAs (*Ab*-miR6, *Ab*-miR7, *Ab*-miR8, *Ab*-miR11, *Ab*-miR15, *Ab*-miR27, *Ab*-miR67) are significantly decreased along the development of CWh leaves, while it is greatly increased in CGr leaves. These miRNAs are predicted to target on genes functioning in DNA repair, translation, sulfate assimilation, starch synthase, oxidation-reduction process, chloroplast development, leaf development, signal transduction etc. These results indicated that these biological processes are different between CGr and CWh leaves and regulated by miRNAs. The expression of most of the significantly differentially expressed miRNAs (|log2 (FC)| ≥ 3 and FDR ≤ 0.01) is down regulated in the CWh leaves. *Ab*-miR39, targeting on SRF-type transcription factor, is differentially expressed not only between CGr and CWh leaves at all three developmental stages, but also among the different developmental stages in the CGr and CWh leaves. SRF-type transcription factor plays roles on the positive regulation of transcription from RNA polymerase II promoter. *Ab*-miR152 is differentially expressed between the CGr and CWh leaves along the development stages. *Ab*-miR152 is a target on transcription factor bHLH18, which regulates transcription. *Ab*-miR124 is significantly down regulated in CWh leaves and at the early development stage of leaves. It is predicted to target on Trihelix transcription factor GT-3b, which may play a role in the induction of CAM4 in response to pathogen and salt^53^. It is predicated that these miRNAs could be presumably important to the albino and development of the leaves and are worth to further studying in the future.

The expression profiles of the nine miRNAs and their potential target genes in the CWh and CGr leaves showed that only a part of the potential target genes are dependent on their miRNAs. It has been previously shown that the expression profiles of all five detected potential target genes are independent of miRNAs in celery^32^. It is believed that the majority of miRNA regulation of genes is indirect^54^ and not all target genes are directly regulated by miRNAs^55^. Previous study has shown that the binding of miRNA is shown to up-regulate the expression of target mRNAs^56^ in certain cellular conditions.

In this study, we reported the first comprehensive study on miRNAs of *A. comosus* var. *bracteatus* performed through high throughput sequencing and bioinformatics analysis methods. Our results showed that there are 163 novel miRNAs being identified, and their potential target genes are predicted and functionally annotated using the Nr, Pfam, KOG/COG, Swiss-Prot, KO, GO and KEGG databases. The miRNAs presumably involved in the albino of the CWh leaves and the development of CGr and CWh leaves were selected and their expression patterns were analyzed. The expression of nine miRNAs related to chlorophyll biosynthesis and photosynthesis and their potential target genes were validated by qRT-PCR. These studies will provide a foundation on elucidating the functions and molecular regulatory mechanisms of miRNAs in the development of normal and albino leaves in *A. comosus* var. *bracteatus*.

## Electronic supplementary material

supplementary information
