# Supplementary material for: High-throughput sequencing analysis revealed the regulation patterns of small RNAs on the development of A. comosus var. bracteatus leaves
Source: Sci Rep. 2018 Jan 31;8:1947. doi: 10.1038/s41598-018-20261-z (PMC5792487; doi:10.1038/s41598-018-20261-z)
Supplement: Supplementary file 1 — supplementary information [file 41598_2018_20261_MOESM1_ESM.pdf]

High-throughput sequencing analysis revealed the regulation patterns of small RNAs on the development of *A. comosus* var. *bracteatus leaves.*

Ying-Yuan Xiong[1], Jun Ma[1]*, Ye-Hua He[2], Zhen Lin[1], Xia Li[1], San-Miao Yu[1], Rui-Xue Li[1], Fu-Xing Jiang[1], Xi Li[1], Zhuo Huang[1], Ling-Xia Sun[1]

[1] College of Landscape Architecture of Sichuan Agricultural University, Chengdu, Sichuan 611100, China.

[2] Horticultural Biotechnology College of South China Agricultural University, Guangzhou, Guangdong 510642, China.

*Corresponding author; E-mail: junma365@hotmail.com

Tel/Fax: +86–28–82652812.

Supplementary Information

SUPPLEMENTARY TABLES

Table S1. Primers of nine miRNAs for reverse transcription and qRT-PCR.

miRNA	Primer for reverse transcription	Sense Primer for qRT-PCR
Ab-miR3	GTCGTATCCAGTGCAGGGTCCGAGGT ATTCGCACTGGATACGACAGCTCT	GTCCGAAGTGCTGTTTGAGT
Ab-miR52	GTCGTATCCAGTGCAGGGTCCGAGGT ATTCGCACTGGATACGACCGTGAG	TCTCGCTCTCCTCTTCTCACG
Ab-miR82	GTCGTATCCAGTGCAGGGTCCGAGGT ATTCGCACTGGATACGACAAGAAG	CCGCTGTACCCTCTCTCTTCTT
Ab-miR108	GTCGTATCCAGTGCAGGGTCCGAGGT ATTCGCACTGGATACGACGCCGCC	TCGTCGGAGGCGGCGT
Ab-miR135	GTCGTATCCAGTGCAGGGTCCGAGGT ATTCGCACTGGATACGACCACCAT	GGTTAGGCTCAGAAGGTATGG
Ab-miR101	GTCGTATCCAGTGCAGGGTCCGAGGT ATTCGCACTGGATACGACCTCCTC	TTGGAAGGGGCATGCAG
Ab-miR11	GTCGTATCCAGTGCAGGGTCCGAGGT ATTCGCACTGGATACGACCTCCTC	TGGAAGGGGCATGCAGAG
Ab-miR125	GTCGTATCCAGTGCAGGGTCCGAGGT ATTCGCACTGGATACGACGAGTCG	GGTGATCTGATAAGAAGCGACT
Ab-miR104	GTCGTATCCAGTGCAGGGTCCGAGGT ATTCGCACTGGATACGACAAGAAG	CTCTCTTCTTGTCGTATCCAGTG
U6		F:GGGGACATCCGATAAAATTGG R:CATTTCTCGATTTGTGCGTGTC

Table S2. Primers of nine potential target genes.

gene	Primer (5'-3')
chlorophyll a-b binding protein of LHCII	F: GATTGGTTGTGGGTTGTG<br>R: TATCCTCTTCTCCTCATCCT
tetrapyrrole-binding protein	F: ACCATCACACACTACAAG<br>R: TCTAAGGAGGAAGAGGAC
PsbP	F: AGAACAAGAACAAGAACAAG<br>R: AAGTAGGCTCTGAACAAC
sedoheptulose-1,7-bisphosphatase	F: TACTTATGTTCTCGCTCTC<br>R: GATTGATGTAGTGTCCTTAAC
ferredoxin-thioredoxin reductase	F: GGACGATCAAGCAGTACG<br>R: ATTCGGATCAGGAACTCG
NADH dehydrogenase	F: AGGAATGGAGGAACTTAATAAC<br>R: GCCCGAGAACATATTGAC
polyphenol oxidase	F: AATAACAACTCCACTCTC<br>R: GTTTGGGACTCTTACAAG
Granule-bound starch synthase	F: GGTCCTTAATCTTAACAACAGTGA<br>R: AGAGCGGTATGCCAATCA
phosphoenolpyruvate carboxylase	F:TATGGTTGGTTACTCTGATTC<br>R: GTCCTCTTGTGCCTTATAC
Histone	F: TATAGCGAAGCATATTGAA<br>R: TTTGGCAGTAAAGTTCTT
18S	F: ATGGTGGTGACGGGTGAC<br>R: CAGACACTAAAGCGCCCGGTA
α-tubulin	F: CCATACAATAGCGTCCTA<br>R: ATAGCCTCGTTATCCAATA

Table S3. Statistics of small RNA sequencing in six samples.

Samples	Raw reads	Containing'N'reads	<18 nt reads	>30 nt reads	Clean reads	Q30(%)
GS1	24213560	0	5432522	2770908	16010130	96.71
GS2	20358050	0	521673	1431317	18405060	96.74
GS3	20037144	0	672333	1534965	17829846	96.92
WS1	20131963	0	2417474	978329	16736160	96.82
WS2	19300504	0	881390	971884	17447230	96.79
WS3	20162392	0	666854	2291470	17204068	96.58

Table S4. Distribution of small RNAs in different categories.

Types	GS1	GS2	GS3	WS1	WS2	WS3
rRNA	7.81%	15.20%	16.68%	17.99%	19.75%	28.60%
scRNA	0.00%	0.00%	0.00%	0.00%	0.00%	0.00%
snRNA	0.00%	0.00%	0.00%	0.00%	0.00%	0.00%
snoRNA	0.07%	0.01%	0.01%	0.04%	0.01%	0.01%
tRNA	2.44%	0.80%	0.89%	1.11%	0.74%	1.35%
Repbase	0.88%	0.07%	0.09%	0.12%	0.08%	0.08%
Unannotated	88.80%	83.91%	82.33%	80.75%	79.42%	69.96%

Table S5. The sequence of the 163 miRNAs detected in *A. comosus* var. *Bracteatus.*

ID	#miRNA	sequence
Ab-miR1	conservative_LG01_1405	TTGGACTGAAGGGAGCTCCT
Ab-miR2	conservative_LG01_21	TCGGACCAGGCTTCATTCCTC
Ab-miR3	conservative_LG01_660	AAGTGCTGTTTGAGTAGAGCT
Ab-miR4	conservative_LG02_3244	TGACAGAAGAGAGTGAGCAC
Ab-miR5	conservative_LG02_3935	ACGGACTGCTGTGATCCTAATAGC
Ab-miR6	conservative_LG02_3947	CTGAAGTGTTTGGGGGAACTC
Ab-miR7	conservative_LG02_3949	CTGAAGTGTTTGGGGGAACTC
Ab-miR8	conservative_LG02_3951	TGAAGTGTTTGGGGGAACTC
Ab-miR9	conservative_LG02_3996	CAGCCAAGGATGACTTGCCG
Ab-miR10	conservative_LG03_5739	TCGGACCAGGCTTCATTCCCC
Ab-miR11	conservative_LG03_5854	TGGAAGGGGCATGCAGAGGAG
Ab-miR12	conservative_LG03_5935	AGTGATCTTGGCTGTGTTGAGTCG
Ab-miR13	conservative_LG04_7077	TGGAGAAGCAGGGCACGTGCA
Ab-miR14	conservative_LG04_7078	TGGAGAAGCAGGGCACGTGCA
Ab-miR15	conservative_LG04_7198	TGAAGCTGCCAGCATGATCTGA
Ab-miR16	conservative_LG04_8194	TCGGACCAGGCTTCATTCCTC
Ab-miR17	conservative_LG04_8801	ATTTGTTGATCGTATCATGTTGTT
Ab-miR18	conservative_LG05_10088	TTGGACTGAAGGGAGCTCCCT
Ab-miR19	conservative_LG05_9023	TGCCAAAGGAGATTTGCCCAG
Ab-miR20	conservative_LG05_9880	TTGACAGAAGATAGAGAGC
Ab-miR21	conservative_LG06_11504	TGGAGAAGCAGGGCACGTGTG
Ab-miR22	conservative_LG06_12427	TGGAGAAGCAGGGCACGTGTG
Ab-miR23	conservative_LG07_12932	TGAAGGACGCAGTAACAAACTATT
Ab-miR24	conservative_LG07_13015	AAGAACTGCTGTACTCCTAATAGT
Ab-miR25	conservative_LG07_13430	GTAGGGATTTGTACTAAAGACGGT
Ab-miR26	conservative_LG07_14572	TTGAGCCGCGTCAATATCTCC
Ab-miR27	conservative_LG08_15328	TGGAAGGGGCATGCAGAGGAG
Ab-miR28	conservative_LG08_15432	TCGGACCAGGCTTCATTCCCC
Ab-miR29	conservative_LG08_15507	TCCGGCTGTTATGCTATCGATAGC
Ab-miR30	conservative_LG09_16236	TGCCTGGCTCCCTGTATGCCA
Ab-miR31	conservative_LG10_18160	TGACAGAAGAGAGTGAGCAC
Ab-miR32	conservative_LG10_18162	TGACAGAAGAGAGTGAGCAC
Ab-miR33	conservative_LG10_18163	TGACAGAAGAGAGTGAGCAC
Ab-miR34	conservative_LG10_18164	TGACAGAAGAGAGTGAGCAC
Ab-miR35	conservative_LG10_18166	TGACAGAAGAGAGTGAGCAC
Ab-miR36	conservative_LG10_18207	TGCCAAAGGAGATTTGCCCAG
Ab-miR37	conservative_LG10_18267	CTTGGATTGAAGGGAGCTCC
Ab-miR38	conservative_LG10_19138	TTGGACTGAAGGGAGCTCCTA
Ab-miR39	conservative_LG10_19250	TGCCAAAGGAGAATTGCCCTG

Ab-miR40	conservative_LG10_19906	AACCTGTTAAGGGGTGGGATACCA
Ab-miR41	conservative_LG11_20645	ACCGATCAAATCTGACATCAAATC
Ab-miR42	conservative_LG11_21011	TTGGACTGAAGGGAGCTCCCT
Ab-miR43	conservative_LG11_21021	TGCCAAAGGAGAATTGCCCTG
Ab-miR44	conservative_LG11_21080	TGACAGAAGAGAGTGAGCAC
Ab-miR45	conservative_LG11_21684	AGCCGGCTTTTGTAGCAAATT
Ab-miR46	conservative_LG11_21960	TGCCAAAGGAGACTTGCCCGG
Ab-miR47	conservative_LG11_21962	TGCCAAAGGAGATTTGCCCAG
Ab-miR48	conservative_LG12_22031	TGCACTGCCTCTTCCCTGGC
Ab-miR49	conservative_LG12_22520	ATGGATTGCTCAGTTTAACGACGG
Ab-miR50	conservative_LG12_22841	TGGAGAAGCAGGGCACGTGCA
Ab-miR51	conservative_LG12_22843	AAGCTCAGGAGGGATAGCGCC
Ab-miR52	conservative_LG12_23566	TCTCGCTCTCCTCTTCTCACG
Ab-miR53	conservative_LG12_23602	TGCCTGGCTCCCTGTATGCCA
Ab-miR54	conservative_LG13_23697	AAGCTCAGGAGGGATAGCGCC
Ab-miR55	conservative_LG13_23700	TGGAGAAGCAGGGCACGTGCA
Ab-miR56	conservative_LG13_23931	GACGTCGATTTGGAGGGCCGTTC
Ab-miR57	conservative_LG13_24414	AGTTACTAATTCATGATCTGGC
Ab-miR58	conservative_LG13_24453	TCCAAAGGGATCGCATTGATC
Ab-miR59	conservative_LG13_24751	GATAGATCTAATGGTGAAAAACTT
Ab-miR60	conservative_LG14_25307	AGAAGTTGTTTTAGAAGCTGGGCC
Ab-miR61	conservative_LG14_25482	TTCGCAGGAGAGATGATGCCG
Ab-miR62	conservative_LG14_25749	TTGAGCCGCGCCAATATCTCT
Ab-miR63	conservative_LG14_25928	TGCCTGGCTCCCTGAATGCCA
Ab-miR64	conservative_LG14_26022	GATAGATCTAATGGTGAAAAACTT
Ab-miR65	conservative_LG14_26031	TGCCTGGCTCCCTGTATGCCA
Ab-miR66	conservative_LG14_26085	TGACAGAAGAGAGTGAGCAC
Ab-miR67	conservative_LG15_26794	TGAAGCTGCCAGCATGATCTGA
Ab-miR68	conservative_LG15_26799	TCGGACCAGGCTTCATTCCCC
Ab-miR69	conservative_LG16_28613	ATTGGATGGATGGGGTGTAAGATG
Ab-miR70	conservative_LG16_29088	AGAAGTAGAAGTTTCAAATTAAAG
Ab-miR71	conservative_LG16_29194	TGACAGAAGAGAGTGAGCAC
Ab-miR72	conservative_LG17_30239	CTAAAGATGGTGCTGAAGGTA
Ab-miR73	conservative_LG18_31927	AGAATCTTGATGATGCTGCAT
Ab-miR74	conservative_LG18_31940	TCCAAAGGGATCGCATTGATC
Ab-miR75	conservative_LG18_32300	GTGATTTGCTGGTAACCTGACAGC
Ab-miR76	conservative_LG19_33150	CTGACAGAAGAGAGTGGGCAC
Ab-miR77	conservative_LG19_33267	TTGAGCCGCGCCAATATCACGT
Ab-miR78	conservative_LG19_33892	GTGTGACAGTGTAGAATTCCT
Ab-miR79	conservative_LG20_34640	TGACAGAAGAGAGTGAGCAC
Ab-miR80	conservative_LG20_34760	TGCCTGGCTCCCTGAATGCCA
Ab-miR81	conservative_LG20_35412	GATGGATGGTTGGTAGGTTGCGGT

Ab-miR82	conservative_LG21_36615	GCTGTACCCTCTCTCTTCTT
Ab-miR83	conservative_LG21_36754	ATTCAAGAAGCATGTAGTATTTGG
Ab-miR84	conservative_LG21_36808	GCAATGGTATCTAATAAGTTTT
Ab-miR85	conservative_LG21_37186	TTAGATGACCATCAACAAACA
Ab-miR86	conservative_LG21_37187	TTAGATGACCATCAACAAACA
Ab-miR87	conservative_LG22_37671	GGGCTTCTCTCTGTTTGGCAGC
Ab-miR88	conservative_LG22_37672	GGGCTTCTCTCTGTTTGGCAGC
Ab-miR89	conservative_LG22_37673	GGGCTTCTCTCTGTTTGGCAGC
Ab-miR90	conservative_LG22_37927	AGAATCTTGATGATGCTGCAT
Ab-miR91	conservative_LG22_38055	GTAGGGATTTGTACTAAAGACGGT
Ab-miR92	conservative_LG23_38768	TGCTCTGTCCGCAGGATTTTCTG
Ab-miR93	conservative_LG24_39753	CCCGCCTTGCACCAACTGAAT
Ab-miR94	conservative_LG24_39803	AGTGCTTCTCTGTTTGCCGCGGTG
Ab-miR95	conservative_LG24_40236	ACGGTCGGTGACATTTTTGTAATC
Ab-miR96	conservative_LG25_40954	TCCACAGGCTTTCTTGAACTG
Ab-miR97	conservative_LG25_40955	TCCACAGGCTTTCTTGAACTG
Ab-miR98	conservative_LG25_41005	AGAATCTTGATGATGCTGCAT
Ab-miR99	conservative_LG25_41161	GATTGGCTTGTTATTGATGACGTG
Ab-miR100	conservative_scaffold_1170_45020	TGCCTGGCTCCCTGAATGCCA
Ab-miR101	conservative_scaffold_1376_45867	TGGAAGGGGCATGCAGAGGAG
Ab-miR102	conservative_scaffold_1613_46696	TTGGACTGAAGGGAGCTCCTA
Ab-miR103	conservative_scaffold_638_42394	ACCGATCAAATCTGACATCAAATC
Ab-miR104	conservative_scaffold_980_44150	GCTGTACCCTCTCTCTTCTT
Ab-miR105	unconservative_LG01_1381	ATTTCGGATTAGCTAACCCCACCT
Ab-miR106	unconservative_LG01_2688	ATATTGGACCTTTTGAGACA
Ab-miR107	unconservative_LG01_2689	ATATTGGACCTTTTGAGACA
Ab-miR108	unconservative_LG01_3	AGATGTATCGTCGTCGGAGGCGGC
Ab-miR109	unconservative_LG01_37	GTAGGAATTTGTACTAAAGACGGT
Ab-miR110	unconservative_LG01_763	ATTTCGGATTAGCTAACCCCACCT
Ab-miR111	unconservative_LG01_798	AGGGCGTCTCTTATAGAACAAGG
Ab-miR112	unconservative_LG02_3879	AAAAACTGCTGTACTCCTAATAGC
Ab-miR113	unconservative_LG02_4026	TTACAAAGACGGTGAACGATTCAC
Ab-miR114	unconservative_LG02_4661	AAGTGTGACAGTGTAGAATTCCTC
Ab-miR115	unconservative_LG02_4662	AAGTGTGACAGTGTAGAATTCCTC
Ab-miR116	unconservative_LG03_5880	TGTTGGTTCGACTCACTCAGG
Ab-miR117	unconservative_LG03_7021	CAGTTTAATATCTGATACGTGGGCC
Ab-miR118	unconservative_LG04_8716	AAAGGCTGCTGTGCTCCTAATAGC
Ab-miR119	unconservative_LG05_10886	TCTTGGGTTCGAATTTAGGAATT
Ab-miR120	unconservative_LG05_10952	AGTAAACGGGAGTGGACATAGCAT
Ab-miR121	unconservative_LG05_9917	TTGGGCCTTCCAGCTAGAAACCT
Ab-miR122	unconservative_LG06_11055	AAGTGCTCTGGCAATATCAATTCC
Ab-miR123	unconservative_LG06_12295	CGTGGATTCGAACATGAGGGCGTG

Ab-miR124	unconservative_LG08_14766	AGTGATGGGAGAATTTTTCTG
Ab-miR125	unconservative_LG09_17100	TGATCTGATAAGAAGCGACTC
Ab-miR126	unconservative_LG09_17859	GTGTCCTTATAATCTAATTCTGTT
Ab-miR127	unconservative_LG10_20027	GGCGGATGTAGCCAAGTGGA
Ab-miR128	unconservative_LG12_22784	ATATTATTAATAAGTCAGCATTGT
Ab-miR129	unconservative_LG12_23098	ATGTGACTTGACGAATGTTGATT
Ab-miR130	unconservative_LG12_23530	AATGGCGGATGGAACGGTCTCACT
Ab-miR131	unconservative_LG12_23592	AACACATAGATTTGCTGGCTCATC
Ab-miR132	unconservative_LG13_23783	GTGCGATGACGGCCGCGCGGGCTCC
Ab-miR133	unconservative_LG13_23938	ACATTATCTAGGTTATTTGAACTT
Ab-miR134	unconservative_LG13_25028	TACTGACTCATCAATAATATTCTG
Ab-miR135	unconservative_LG14_25402	TTAGGCTCAGAAGGTATGGTG
Ab-miR136	unconservative_LG14_25403	TTAGGCTCAGAAGGTATGGTG
Ab-miR137	unconservative_LG14_25604	AAATGATCTGAAGTTGAATGTGTC
Ab-miR138	unconservative_LG14_25863	CATGATGTCGACTAATAATAT
Ab-miR139	unconservative_LG15_26845	ATTGGCAATATACGCTATTCTGAGA
Ab-miR140	unconservative_LG15_27619	TTACGGTTTGTAAGTAGTGAGC
Ab-miR141	unconservative_LG15_27794	TAAGTTGAACACGGTGAACCATTC
Ab-miR142	unconservative_LG15_27889	TGCACTAATGTGAAGGAAATTGTAG
Ab-miR143	unconservative_LG16_28851	CTGTAAACCGCACGACTCTTC
Ab-miR144	unconservative_LG16_29930	CTGCCAAAGGAGATTTGCCCC
Ab-miR145	unconservative_LG17_30219	GTTGAAGTATGAGATGAGATGGAT
Ab-miR146	unconservative_LG19_32802	AAATGCTATTATTTGGACGGT
Ab-miR147	unconservative_LG19_33497	AAATGCTATTATTTGGACGGT
Ab-miR148	unconservative_LG20_34716	TAGATGTTGTAGATCATATCTATC
Ab-miR149	unconservative_LG21_36012	AAAGAGTGGTAGAAAGGACTATA
Ab-miR150	unconservative_LG21_36022	AAAAGATCTCTTGGAATATTGTTG
Ab-miR151	unconservative_LG21_36397	TAAGTTGAACACGGTGAACCATTC
Ab-miR152	unconservative_LG21_37127	TAAGGATCTACTGTTGAACAGAGT
Ab-miR153	unconservative_LG22_37439	AAAAACGGTCTATAGCTACACTTT
Ab-miR154	unconservative_LG22_38657	TTGACTGACTGGTCTTTCTCTTTAG
Ab-miR155	unconservative_LG24_40567	AAGTGGTTGTAGAGTTAGCATATC
Ab-miR156	unconservative_LG25_40921	TCGACTGTCCCTAGAGCAAGTGGC
Ab-miR157	unconservative_LG25_41071	GTTAGCACGTGACTGTCACTTGAC
Ab-miR158	unconservative_LG25_41192	TCAATGCGATTTCTCTGGAAT
Ab-miR159	unconservative_scaffold_1066_44611	ATTCGAACTTGCAGACTCGTTGCA
Ab-miR160	unconservative_scaffold_1511_46357	GGCGGATGTAGCCAAGTGGA
Ab-miR161	unconservative_scaffold_1590_46617	GGCGGATGTAGCCAAGTGGA
Ab-miR162	unconservative_scaffold_393_41527	CCGGATATACGCATTGGACACGGC
Ab-miR163	unconservative_scaffold_864_43667	GGCGGATGTAGCCAAGTGGA

Table S6. Statistics of the functional annotation of potential target genes.

Anno database	Anno number	300≤length<1000	Length≥1000
COG_Annotation	155	26	128
GO_Annotation	243	67	174
KEGG_Annotation	148	39	107
KOG_Annotation	228	55	172
Pfam_Annotation	363	87	273
Swissprot_Annotation	320	82	235
Nr_Annotation	414	112	298
All Annotated	418	113	300

SUPPLEMENTARY FIGURES

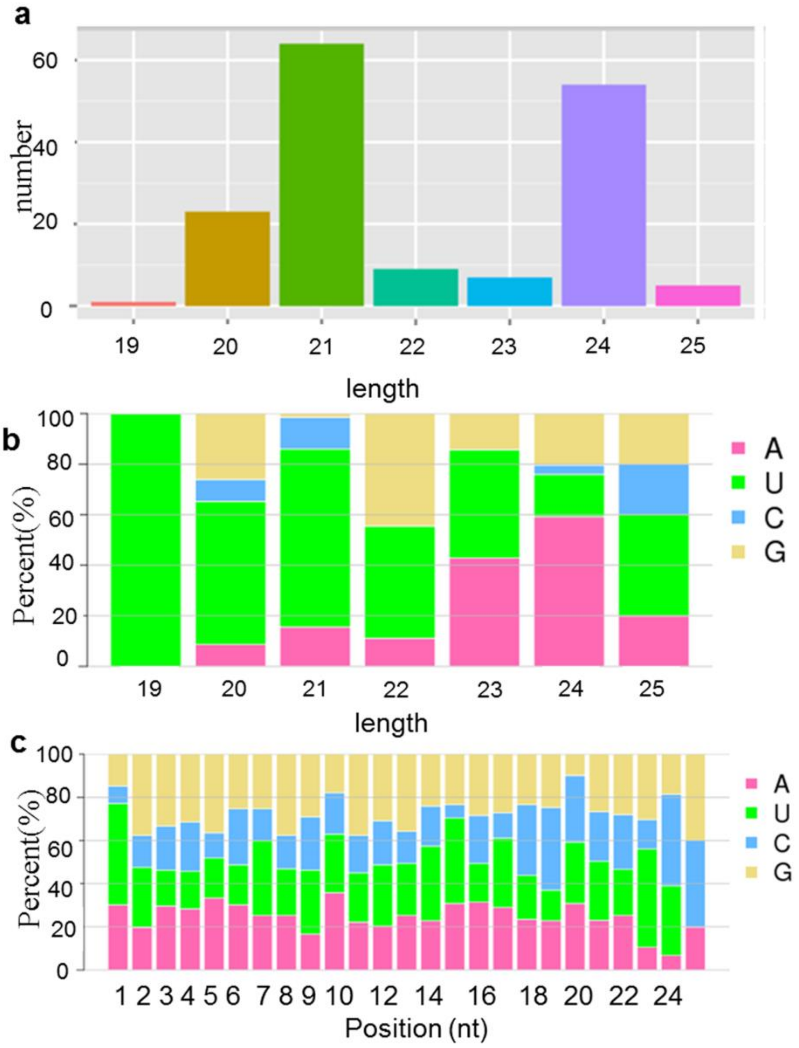

Figure S1. Length distribution and base bias analysis of miRNAs detected in six samples. (**a**) Length distribution of miRNAs. (**b**) Analysis of nucleotide bias percentage at miRNA first nucleotide bias in miRNAs. (**c**) Analysis of nucleotide bias percentage at each position in miRNAs.

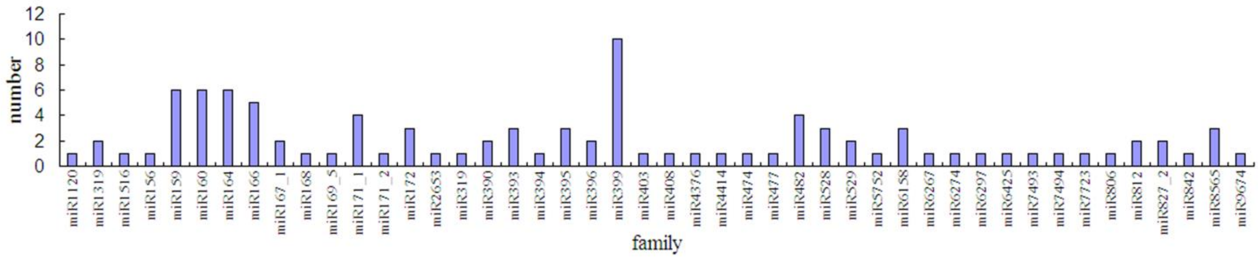

Figure S2. Distribution of identical miRNA members in each family. Small RNA sequences were compared with currently known miRNAs in the miRbase database.

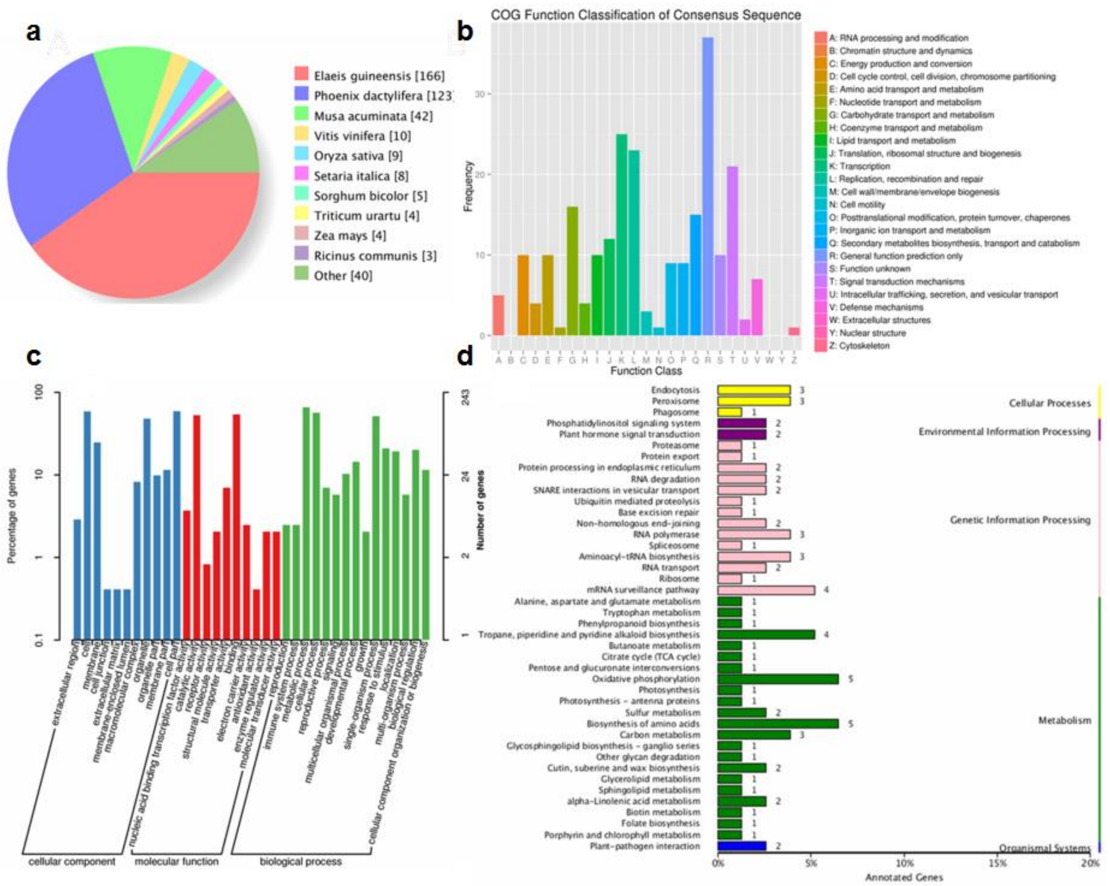

Figure S3. Annotation analysis of the miRNAs. (**a**) The Nr homologous species distribution of the candidate target genes. (**b**) Classification annotation of miRNA target genes based on COG database. (**c**) GO classification of miRNA target genes. (**d**) KEGG classification of miRNA target genes.
